# Stimulus Contrast Information Modulates Sensorimotor Decision Making in Goldfish

**DOI:** 10.3389/fncir.2020.00023

**Published:** 2020-05-28

**Authors:** Santiago Otero Coronel, Nicolás Martorell, Martín Beron de Astrada, Violeta Medan

**Affiliations:** ^1^Department Fisiología y Biología Molecular y Celular, Facultad de Ciencias Exactas y Naturales, Universidad de Buenos Aires, Buenos Aires, Argentina; ^2^Instituto de Fisiología, Biología Molecular y Neurociencias (IFIBYNE), CONICET-Universidad de Buenos Aires, Buenos Aires, Argentina

**Keywords:** goldfish, stimulus contrast, C-start, defensive behavior, stimulus saliency

## Abstract

Animal survival relies on environmental information gathered by their sensory systems. We found that contrast information of a looming stimulus biases the type of defensive behavior that goldfish (*Carassius auratus*) perform. Low-contrast looms only evoke subtle alarm reactions whose probability is independent of contrast. As looming contrast increases, the probability of eliciting a fast escape maneuver, the C-start response, increases dramatically. Contrast information also modulates the decision of when to escape. Although response latency is known to depend on looming retinal size, we found that contrast acts as an additional parameter influencing this decision. When presenting progressively higher contrast stimuli, animals need shorter periods of stimulus processing to initiate the response. Our results comply with the notion that the decision to escape is a flexible process initiated with stimulus detection and followed by assessment of the perceived risk posed by the stimulus. Highly disruptive behaviors as the C-start are only observed when a multifactorial threshold that includes stimulus contrast is surpassed.

## Introduction

Evasive behaviors are essential to avoid harm from predators or other threats in the environment. Although critical for animal survival, escaping comes at the cost of interrupting other behaviors such as foraging or mating, and thus it is not performed unless the perceived threat surpasses a decision threshold (Lima and Dill, 1990). To match behavior to perceived risk animals first detect and then evaluate threat levels to decide whether to escape.

One of the best studied escape behaviors is the C-start of fish (Dill, 1974; Eaton, 1984; Batty, 1989; Faber et al., 1989; Preuss and Faber, 2003; Kohashi and Oda, 2008; Neumeister et al., 2010). The C-start is a high-threshold escape behavior consisting of a first stage where fast and massive unilateral contraction of trunk muscles results in the fish adopting a C-shape followed by a return stroke in the opposite direction (“return flip”) where the tail straightens propelling the animal away from the potential danger (Domenici, 1997; Eaton et al., 1977; Zottoli, 1977). Although the initial stage is highly stereotyped, and its directionality is mostly imposed by the direction of the threat, it can be modulated by the presence of obstacles or other fish (Eaton and Emberley, 1991; Domenici, 2010).

However, before deciding to execute an evasive behavior, fish have to evaluate threat levels. Perceived threat levels might be strong enough to trigger an immediate escape reaction as the C-start, but if the information is ambiguous or if there is no actual interaction between the sources of threat (predator) and the animal, an alarm reaction might be the most adaptive response (Lima and Dill, 1990; Smith, 1992). Fish can display a variety of alarm reactions that not only decrease the risk for the alarmed individual but also might signal the presence of danger to conspecifics (Brown et al., 1999; Oliveira et al., 2017). These alarm responses include spine erection and fin flicking (Brown et al., 1999), rapid swimming into a hiding place (if there is one), darting, erratic swimming, reduced activity, or freezing (Pfeiffer, 1962; Smith, 1992; Kalueff et al., 2013).

The level of perceived risk posed by a stimulus will depend on its specific characteristics as well as internal state and prior experience of the animal (Magurran, 1990; Brown and Smith, 1996; Brown et al., 2016). Escape thresholds can rise when animals are feeding or when previous encounters with the stimulus had no harmful consequences (Lima and Dill, 1990; Roberts et al., 2016, 2019; Lloyd and Dayan, 2018). Vigilance levels can also affect threat detection (De Franceschi et al., 2016). For example, in fish that are actively exploring the environment, threat detection can produce an interruption of ongoing locomotion to stabilize the visual panorama and facilitate tracking the stimulus.

In laboratory conditions, robust escape behavior can be elicited by visual looming threats (Eaton et al., 1981; Preuss et al., 2006; Temizer et al., 2015; Dunn et al., 2016). Looming stimuli usually consist of computer-generated black disks rapidly expanding over a white background. This type of stimuli has been shown to induce escape behaviors from invertebrates to humans (Laurent and Gabbiani, 1998), suggesting that the neural circuits involved in avoidance of an approaching predator or a collision have evolved early during evolution (Evans et al., 2019). Fish can compute looming velocity and retinal angular size to decide when to initiate a C-start (Preuss et al., 2006; Temizer et al., 2015; Dunn et al., 2016; Heap et al., 2018) and to adjust the kinematics of the escape swim (Bhattacharyya et al., 2017). However, to our best knowledge, the effect of decreasing the contrast between the looming and the background on C-start kinematics has not been explicitly tested. Here, we varied the contrast of looming stimuli to manipulate its salience and investigated the effect on the behavioral choices fish performed. In addition, we tested the hypothesis that stimulus contrast is incorporated in the computing mechanism that determines C-start response latency.

## Methods

### Animals

Adult goldfish (*Carassius auratus*) of both sexes, 7–10 cm of standard body length, were purchased from FunFish (Córdoba, Argentina). Fish were allowed to acclimate for at least a week after transport and were kept in rectangular glass holding tanks (30 × 60 × 30 cm; 95 L) in groups of 10 animals. Tanks were supplied with filtered and dechlorinated water and maintained at 18°C. Ambient light was set to a 12-h light/dark photoperiod. Animals were fed floating pellets (Sera, Germany) five times a week.

All animal procedures were performed in accordance with the guidelines and regulations of the Institutional Animal Care and Use Committee of Facultad de Ciencias Exactas y Naturales, Universidad de Buenos Aires (protocol #70).

### Experimental Setup and Behavioral Protocol

**Figure 1:**
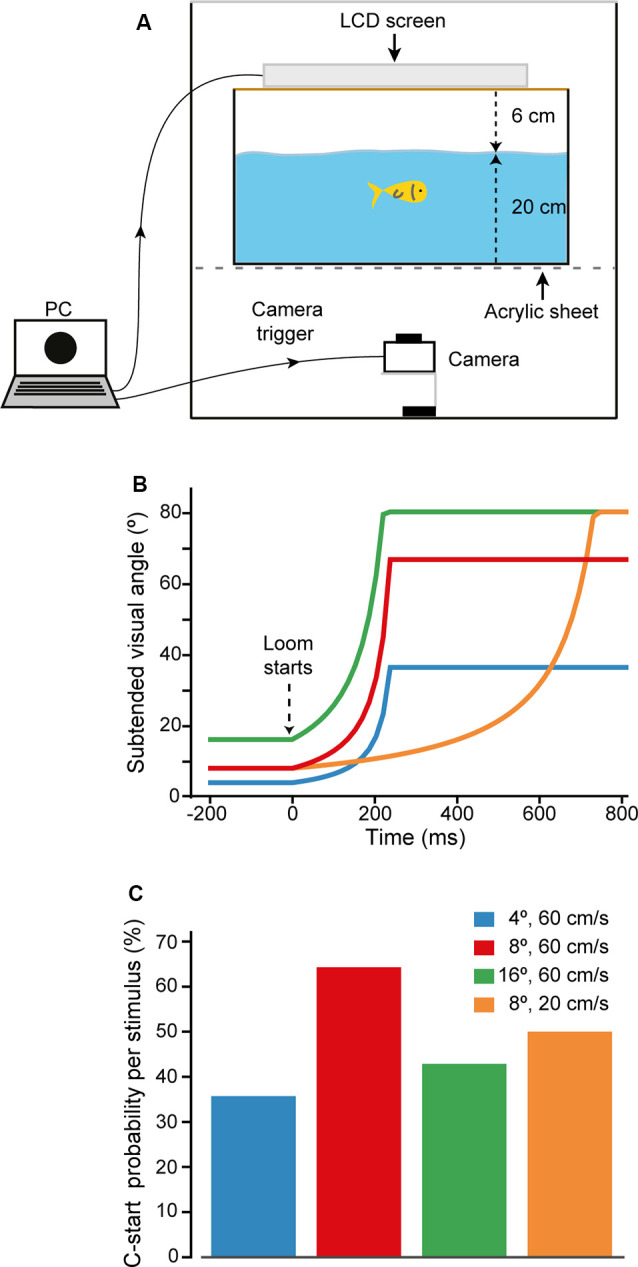
Looming evoked escapes in goldfish. **(A)** Behavioral setup. Computer-generated visual looms were delivered through an LCD screen located on top of the experimental aquarium, which stands on a transparent acrylic platform. Fish behavior and looming expansion were filmed from below at 240 or 480 fps. The same computer triggered acquisition of the camera. **(B)** Time course of the stimuli used to characterize response probability to high contrast visual looms. Stimuli differed in their initial subtended angle (4°, 8°, or 16°) or expansion velocity (20 or 60 cm/s). **(C)** C-start probability for each of the looms used. After this initial characterization, the stimulus that evoked maximal C-start probability, loom onset at 8° and expanding at 60 cm/s (red), was chosen for the rest of the experiments.

Goldfish were tested in a rectangular experimental tank (48-cm length, 36-cm width, and 27-cm height) with its external walls covered with black opaque cardboard to avoid external visual stimulation. In addition, opaque panels covered all sides and top of the experimental setup, preventing external light to reach the tank. Experiments were made in a silent room with ceiling lights off. The experimental tank was filled with filtered dechlorinated water up to a height of 20 cm. A liquid crystal display (LCD) screen used for visual stimulation was secured 6 cm above the water surface (Figure 1A). The long axis of the screen was placed parallel to the long axis of the tank. Illumination was homogeneous throughout the tank, and no shelters were provided. The tank was situated on a transparent acrylic sheet, allowing video recording of the fish’s behavior and stimulus presentation from beneath at 240 or 480 fps (Casio EX ZR100, Tokyo, Japan).

Computer-controlled presentation of visual stimuli on the LCD screen and triggering of the camera acquisition occurred 1.3 s before the stimulus appeared and stopped at 9.7 s after the end of visual stimulation. A web camera recorded fish activity (60 fps) from below and allowed us to monitor animal activity during the experiment.

Individual fish were placed in the experimental tank and allowed to acclimate for 30 min. Unless otherwise stated, the animal was then stimulated three times with the same looming stimulus with a 5-min interval between presentations. The small number of stimulus presentations and the relatively long intertrial intervals were chosen to minimize habituation effects (Gallagher and Northmore, 2006; Temizer et al., 2015). After the experiment, the animal was returned to its holding tank.

### Visual Stimuli

Computer-generated black disks that expand over a white background (loom) efficiently elicit C-start escapes (Preuss et al., 2006; Medan and Preuss, 2014; Temizer et al., 2015; Dunn et al., 2016). In our study, looms were presented using a 380 × 305 mm LCD (1,280 × 1,024 pixels, refresh rate 75 Hz, HP L1940T; Hewlett-Packard, Palo Alto, CA, USA). While keeping the background white (i.e., all three values in the RGB code set to 255), we modified the grayscale value of the disk stimulus (all RGB values set to either 0, 225, 235, 240, 245, 249, or 253) to obtain various intensity contrast (IC) stimuli. To characterize the luminance of each component of our stimuli, we used an irradiance sensor (J1812) of a Tektronix J17 photometer (Wilsonville, Oregon, MI, USA) positioned in the center of the tank. During these measurements, all pixels in the screen were set to the RGB value that we were currently testing. The RGB triplets yielded intensities ranging from 19.9 mW/m^2^ (RGB values set to 0) to 338.5 mW/m^2^ (RGB values set to 255).

We used these irradiance measurements to determine the IC for each stimulus, calculated as the Michelson index (%), where contrast is defined as (*I*_disk_ − *I*_bkgn_) × 100/(*I*_disk_ + *I*_bkgn_), and *I*_disk_ and *I*_bkgn_ refer to the irradiance of the expanding disk and the background, respectively. The contrast of the different IC looms will be subsequently denoted by their subindex (e.g., IC_1.7_ represents an IC with a Michelson index of 1.7%).

Although it has been repeatedly shown that overall decreases in luminance (dimming stimuli) do not evoke C-starts, escape responsiveness in fish does depend on the dynamics of the visual loom stimulus (Preuss et al., 2006; Burgess and Granato, 2007; Temizer et al., 2015; Dunn et al., 2016). Therefore, an ineffective stimulus dynamic could represent a confounding factor when trying to detect contrast sensitivity with a looming stimulus. To ensure that our stimulus dynamic was efficiently triggering escape responses, we tested ICs in which black disks expanded over a white background with four different dynamics (Michelson contrast 89%, Figure 1B). To calculate the angular retinal size of the disk, we assumed the fish to be in the center of the tank at the midpoint of the water column (Figure 1A). Three of the four stimuli simulated the approach of a sphere at 60 cm/s that subtended an angle of 4°, 8°, or 16° at its stationary initial position and expanded up to 37°, 67°, or 80°, respectively, in 221 ms (Figure 1B). The fourth stimulus simulated a sphere moving at 20 cm/s that initially subtended an angle of 8° and reached 80° in 731 ms. Each of the four loom dynamics was tested twice in random order on each animal with an intertrial interval of 5 min (Figure 1C). We found that although all stimuli were effective, the stimulus that subtended an initial angle of 8° and a velocity of 60 cm/s provoked the C-start response with highest probability (64%, *N* = 7, *n* = 14, Figure 1C, red bar). We, therefore, used this dynamic for the rest of the experiments.

### Data Analysis

#### Behavioral Responses

##### C-start Escape Responses

Videos were analyzed offline using ImageJ (National Institutes of Health, Bethesda, MD, USA). Visual inspection of the videos allowed us to confirm the initial scoring of occurrence of C-start escape responses observed during the experiment and to measure its latency with ±2 ms error for videos recorded at 480 fps and ±4 ms error for videos recorded at 240 fps. The first frame at which the expanding loom attained its maximum size was considered as 0 ms. Therefore, C-start responses occurring before the end of the expansion have a negative latency, whereas those occurring after rendered a positive latency.

##### Alarm Responses

Videos were also inspected and scored by three independent observers to analyze the occurrence of behaviors other than C-start responses. These responses included behaviors suggesting increased arousal and alarm. Alarm responses consist on a variety of subtle but robust motor reactions including accelerating or decelerating swimming, darting (a single fast acceleration in one direction with the use of the caudal fin), erratic movements/zigzagging (representing fast acceleration bouts in rapid succession), and rapid abduction of fins with no body displacement (Savage, 1971; Laming and Savage, 1980; Brown et al., 1999; Kalueff et al., 2013). An alarm response was computed when scoring of occurrence and description of the behavior matched for all observers.

### Statistical Analysis

R (version 3.6.1, www.r-project.org) and R Studio (version 3.5.0, rstudio.com) were used for statistical analysis. A significance level of *α* = 0.05 was used throughout the study. The effect of looming contrast on the probability of executing alarm responses or an escape behavior was assessed with a binomial generalized linear model (GLM) considering contrast levels as a fixed factor. The effect of varying contrast on latency was analyzed with a linear model. Sample size is denoted by *N* when it refers to the number of animals or *n* when it refers to the number of trials.

## Results

**Figure 2:**
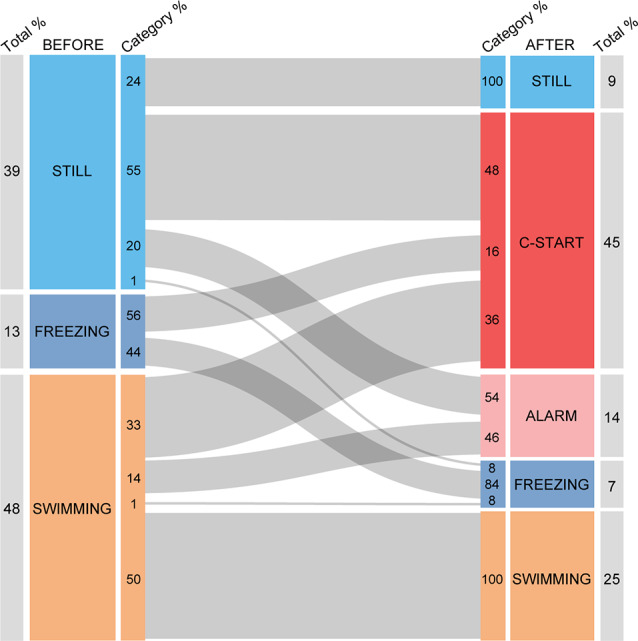
Alluvial diagram of motor behavior before and after looming stimulation. The diagram shows the motor animal’s motor state before (left) and after (right) looming stimulation. Numbers next to the outer edge of the boxes indicate the proportion of animals in each category. Numbers next to the inner edge of the boxes indicate the proportion of animals within each category transitioning from one type of behavior to another. Behavior after stimulation is significantly dependent on previous motor state (*χ*^2^ test of homogeneity, *p* < 0.001).

As behavioral decisions are influenced by the immediate behavioral past Magurran (Magurran, 1990; O’Connor et al., 2015; Stephenson, 2016; Schaerf et al., 2017), we first analyzed whether an animal’s behavior before looming expansion modulates its response to the stimulus. For this analysis, we included IC stimuli that evoked C-starts and alarm responses in at least 30% of the animals (ICs ranging from 89% to 2.8%, *n* = 197, *N* = 68) to reduce the probability of including trials in which fish did not detect the stimulus. We classified the prior motor state of fish in three categories: (1) still, referring to animals that were only moving the pectoral fins with no net displacement of the body; (2) freezing, when we could detect no movement other than breathing; and (3) swimming, when fish were actively moving the caudal fin and producing a net propulsion of their body. We then analyzed the transitions from those three prestimulation states to the different behavioral outcomes of the looming stimulation. The behaviors observed after looming presentation included those mentioned before (Categories 1–3) and, in addition, C-start and visual alarm responses (see “Methods” section). Although freezing might be considered an alarm response (Kalueff et al., 2013), we computed it as a different category because, in contrast with the rest of alarm responses, some animals were already freezing at the time of stimulation. The alluvial diagram of Figure 2 depicts how the previous motor state of fish affects the probability distribution of behavioral responses observed after stimulation. Behavior after stimulation is significantly dependent on previous motor state (*χ*^2^ test of homogeneity, *p* < 0.001). The diagram shows that 45% of all stimulations evoked a C-start, and two-thirds of these responses (64%) were produced by animals that were either still (48%) or freezing (16%) before the expansion, and only 36% of the responses correspond to animals that were swimming prior to the expansion. This suggests that being still might aid stabilizing visual panorama and therefore improve threat detection, or it could also be indicative of higher vigilance, two factors that could increase C-start probability. Consistent with this idea, 50% of animals that were swimming when the looming occurred did not change their behavior. The results also show that freezing does not preclude by itself the execution of an explosive response as the C-start as more than half (56%) of the animals that were freezing responded with a C-start. On the other hand, we never observed a transition from freezing to another type of alarm response or a transition from immobility (still) to swimming.

In each of the three prestimulation behavioral categories defined, a proportion of animals did not modify their previous behavior. This could be either the result of animals detecting the stimulus and deciding not to alter their behavior or simply the failure to detect the stimulus. In particular, animals that were freezing could have detected the stimulus and judged it threatening but decided that freezing was the best response. Actively swimming animals, which in the majority of cases (50%) did not change their behavior, could have similarly failed to detect the stimulus. Alternatively, they may have judged interruption inadequate as they were already engaged in another activity (e.g., exploring the arena for shelter or an exit). Our behavioral analysis is insufficient to distinguish between these options.

Because our behavioral protocol included three consecutive presentations of the same looming stimulus, we analyzed if each animal showed consistent responses across trials or if the behavioral choice changed, for example, due to habituation. The response during the first trial did not affect the chance of the same type of behavior recurring, resulting in no consistent pattern of response at the individual level (Supplementary Figure S1). This result suggests that each response was independent of the previous one, although a different stimulation protocol (e.g., shorter intertrial periods or more stimulus presentations) may lead to different results. The overall proportion of behaviors observed for each trial was similar (Supplementary Figure S2, *χ*^2^ test, *p* = 0.86), that is, the same proportion of animals (but not necessarily the same individuals) performed each type of behavior.

**Figure 3:**
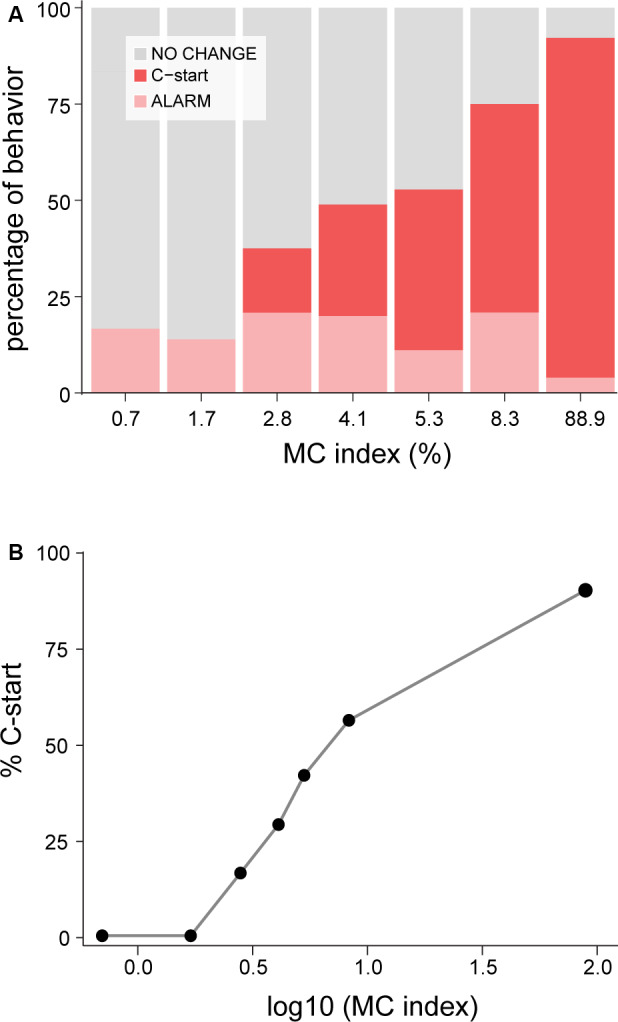
Increasing contrast shifts fish response to C-start behavior. **(A)** Stacked bars indicate the relative proportion of C-starts (red), alarm responses (pink), or no change (gray) in motor behavior. Alarm behaviors are grouped but include decelerations, accelerations, and fin abductions, whose proportions did not change across stimuli. Higher contrast stimuli produced a higher proportion of C-starts (binomial generalized linear model (GLM), effect of contrast on C-start probability, *p* < 0.001) but no significant change on the proportion of alarm responses (binomial GLM, effect of contrast on alarm responses, *p* = 0.03). **(B)** Percentage (%) of C-start probability vs. MC represented in a logarithmic scale.

After an evaluation period and depending on the risk perceived, fish will ignore the stimulus if no risk is detected, perform an alarm reaction if the perceived danger is intermediate, or opt for a last-resource evasive behavior when danger is extreme. To test if increasing the saliency of the stimulus modulates this decision making, we analyzed whether looms of increasing IC (see “Methods” section) modified the proportion of these behaviors (Figure 3A). We found that although all looming stimuli had identical duration and identical subtended angle, increasing contrast produced a gradual switch from no evident motor reaction for looms of IC_0.7_ (83% did not alter their behavior, *n* = 36) to an almost exclusive election of C-start escape for IC_89_ (90% performed C-starts, *n* = 49; binomial GLM on the effect of contrast on C-start probability, *p* < 0.001). Threshold contrast for C-starts lies between IC_1.7_ and IC_2.8_ and follows the Weber–Fechner law, as evidenced by the linear increase when the Michelson contrast is represented on a logarithmic scale (Figure 3B). This is, to our best knowledge, the first evidence that goldfish incorporate the level of looming contrast when computing its threatening value.

Curiously, alarm responses show no modulation by contrast as they were equally probable (between 11% and 20%, Figure 3A, light pink bars) for all ICs except for IC_89_, where only 4% of fish produced an alarm response (binomial GLM on the effect of contrast on the proportion of alarm responses, *p* = 0.03). Despite the relatively low number of alarm responses to each IC, the proportion of each class of alarm behavior did not seem to change across ICs, with an average of 33% of the alarm behaviors corresponding to accelerations, 55% to decelerations, and 12% to rapid abductions of fins (Supplementary Figure S3, *χ*^2^ test, *p* = 0.48).

**Figure 4:**
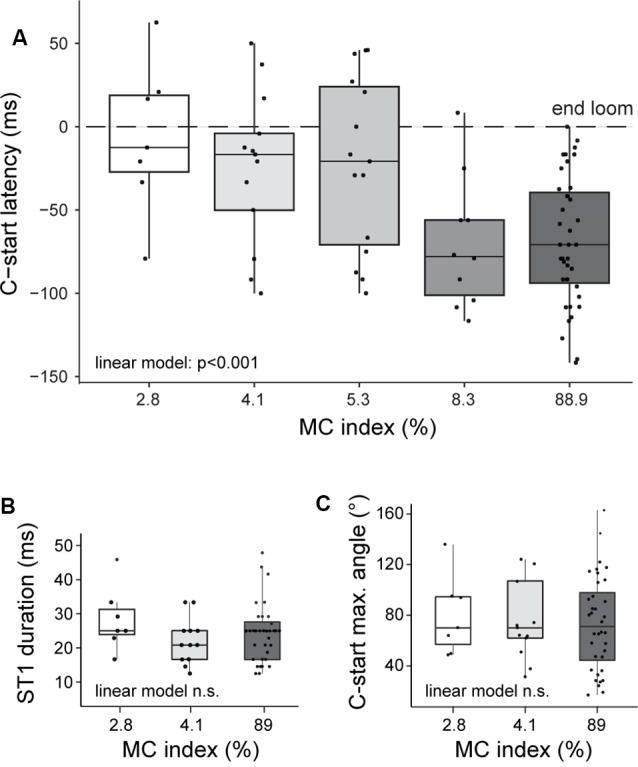
Stimulus saliency is correlated with C-start response latency but does not change its kinematics. **(A)** Looming stimuli of increasing Michelson index were associated with shorter response latencies (linear model, effect of contrast on latency, *p* < 0.001). Stage 1 duration **(B)** or maximum stage 1 angle **(C)** is similar for IC_2.8_, IC_4.1_, and IC_89_ (linear model, effect of contrast on duration, *p* = 0.77; effect on angle, *p* = 0.70). Boxplots represent median and 25th and 75th quartiles and minimum and maximum values for each MC. Superimposed dots represent individual responses.

Because all the stimuli used in our experiments had the same expansion dynamics, we expected a fixed C-start latency (Bhattacharyya et al., 2017). Surprisingly, we found that goldfish progressively delay their escapes when saliency was lowered; that is, they took progressively more time to initiate the C-start [Figure 4A, linear model on the effect of contrast on latency, *p* < 0.001, Shapiro test not statistically significant (n.s.)]. While the highest-contrast looms produced mean (±SD) latencies of approximately 70 ms before the end of the loom expansion (IC_89_: 69 ± 38 ms, IC_8.3_: 71 ± 39 ms), lower-contrast looms delayed response by about 50 ms (IC_2.8_: 7 ± 45 ms; IC_4.1_: 25 ± 46 ms before the end of the expansion). In fact, lower saliency looms evoked a significant proportion of C-starts that occurred after the end of the expansion (43%for IC_2.8_).

We also analyzed if other parameters of the C-start response were affected. We compared the kinematics of C-starts evoked by high and low IC (IC_89_, IC_2.8_, and IC_4.1_) corresponding to 56 trials performed in 30 animals. If low- and high-contrast looms were recruiting the same sensorimotor networks, we expected the main characteristics of stage 1 of the C-start (initial bending producing a C-shape that precedes the return flip) to be similar across different ICs. Indeed, animals startling to low- or high-contrast looms did not differ in C-start duration (Figure 4B, median, IC_89_: 25 ms; IC_4.1_: 21 ms, IC_2.8_: 25 ms, linear model n.s.) or angular bend performed (Figure 4C, median, IC_89_: 71°; IC_4.1_: 70°, IC_2.8_: 70°, linear model n.s.). Although these results do not exclude the possibility that other reticulospinal neurons are implicated, they suggest that C-starts evoked by high- or low-contrast looms are conveyed through similar networks.

## Discussion

Although fast defensive behaviors such as startle were initially analyzed as reflex-like responses organized around command neurons (Edwards et al., 1999; Eaton et al., 2001), further investigation has revealed a deeper complexity and flexibility than previously acknowledged (Domenici, 2010; Simmons et al., 2010; Card, 2012; Evans et al., 2019).

Studies on defensive responses in fish have mainly centered on the C-start escape response and the parameters of the auditory or visual stimuli that trigger this response. Our experiments using less salient stimuli reveal a wider behavioral flexibility of the defense response at two levels. First, we found that fish can display a range of behaviors that include C-starts but also a variety of alarm responses. Second, we found that if a C-start is performed, the time to initiate it is modulated by the contrast of the stimulus.

When reducing the disk luminance of an otherwise invariant looming stimulus, we observed that C-start probability decreased until disappearing for contrasts lower than IC_2.8_. Dimming stimuli were previously shown to be ineffective to elicit high-velocity escapes when compared to checkered loomings with constant luminance (Temizer et al., 2015; Dunn et al., 2016). Thus, the observed effects on C-start probability of our study cannot be explained by differences in final total luminance across stimuli. Instead, we postulate that the higher contrast between disk and background is the key variable responsible for increasing the detectability and saliency of the stimulus and thus the C-start probability.

Additionally, we observed a rather constant rate of alarm behaviors that only decreased for the highest-contrast loom. This suggests that the initial process of stimulus detection is, above a minimum sensitivity threshold, contrast independent for the range between IC_0.7_ and IC_8.3_. Our study suggests that contrast does not affect the probability or type of alarm behaviors, although this result should be regarded with caution given the relatively low number of alarm responses obtained for each IC stimulus. It could be possible that a reduction on alarm responses when contrast is reduced might be detectable only if more sensitive techniques are implemented. Heart rate frequency or skin conductance response levels are traditionally used to measure “alarm” (Burnovicz et al., 2009; Yoshida et al., 2009; Kreibig, 2010).

In addition to modulating C-start probability, looming contrast also modulates *when* animals initiate the C-start. C-start response probability has been shown to be modulated by external factors such as spatial and social context (Eaton and Emberley, 1991; Fischer et al., 2015) and internal state variables such as hunger level or reproductive or social status (Neumeister et al., 2010; Filosa et al., 2016; Park et al., 2018). Furthermore, intrinsic characteristics of the stimulus such as its modality, temporal dynamics, or directionality have also been shown to shape the characteristics of the C-start. Specifically for visual looms, the relationship between C-start latency, loom velocity, and subtended angle at the retina has been extensively studied (Preuss et al., 2006; Temizer et al., 2015; Dunn et al., 2016; Bhattacharyya et al., 2017). On the other hand, the dependency of the C-start with the contrast of the stimulus has only been previously investigated by presenting black-over-white or white-over-black combinations (Medan and Preuss, 2014; Temizer et al., 2015; Dunn et al., 2016; Randlett et al., 2019). In nature, however, animals are exposed to a continuous scale of contrast between objects and background. Here, we found that, indeed, animals take into account the contrast to assign salience to a looming stimulus. If contrast is interpreted as a source of information, then high-contrast looms might provide more salient information, allowing to reach the decision threshold in a shorter period of time (Portugues et al., 2015; Bahl and Engert, 2020). As looming contrast diminishes, animals may need to integrate visual information for longer periods before reaching the C-start threshold, producing the increase in latency we observe.

Heap et al. (2018) have recently proposed that visual information is conveyed by the retina not only through the optic tectum but also through the thalamus. They observed that thalamotectal projection neurons modulate the responses of looming sensitive tectal neurons. Luminance information carried by thalamic projection neurons increased C-start response rate and was found necessary to evoke directional escapes. While that study did not vary luminance contrast, it would be reasonable to expect that luminance contrast correlates with the strength of thalamic input to the tectum. This, in turn, would possibly lead to stronger input to downstream reticulospinal networks resulting in escapes with shorter latency, providing a mechanistic basis to the correlation we obtained between contrast and response latency.

The behavioral flexibility observed for less salient stimuli has been attributed to activity in different sets of tectal neurons, which in turn innervate different populations of spinal projecting nuclei (Bhattacharyya et al., 2017). This is paralleled by the flexibility described for C-start escapes, which depend on the activation of different reticulospinal neurons that in conjunction with the Mauthner cell are collectively known as the brainstem escape network (Eaton et al., 2001; Gahtan et al., 2002; Canfield, 2003; Weiss et al., 2006). Whether neural circuits subserving alarm responses and the C-start behavior are different or at least partially overlapping awaits further investigation.

## Data Availability Statement

The datasets generated for this study are available on request to the corresponding author.

## Ethics Statement

The animal study was reviewed and approved by Institutional Animal Care and Use Committee of Facultad de Ciencias Exactas y Naturales, Universidad de Buenos Aires.

## Author Contributions

VM and MB contributed to conception of the study. SO and VM participated in the design of the experiments, carried out experiments, and performed data analysis. NM performed experiments. SO, MB, and VM contributed to drafting and revising the manuscript and all authors approved the final version of the manuscript.

## Conflict of Interest

The authors declare that the research was conducted in the absence of any commercial or financial relationships that could be construed as a potential conflict of interest.
