# Supplementary figures and images for: Stimulus Contrast Information Modulates Sensorimotor Decision Making in Goldfish
Source: Front Neural Circuits. 2020 May 28;14:23. doi: 10.3389/fncir.2020.00023 (PMC7270408; doi:10.3389/fncir.2020.00023)

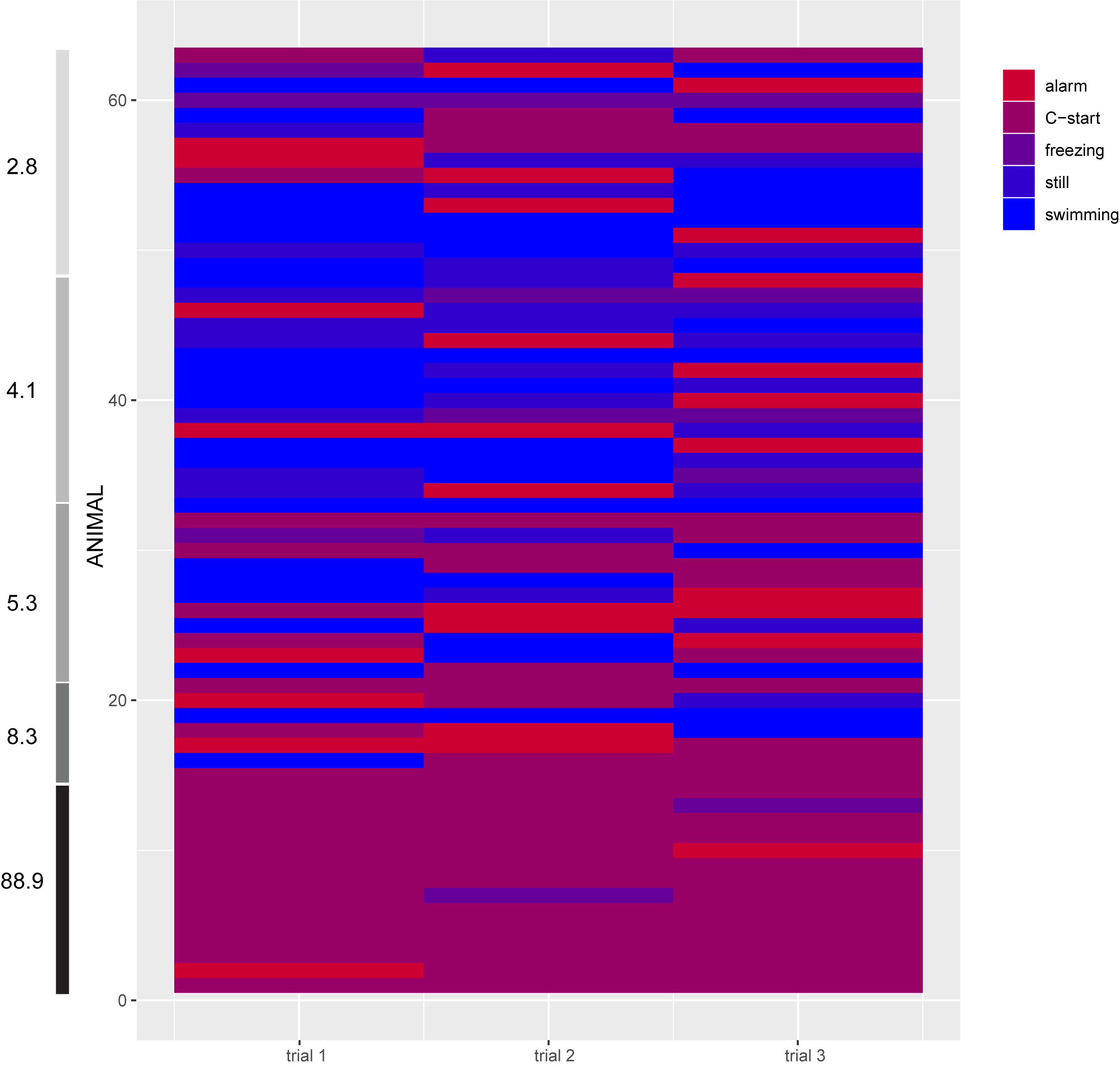

Supplement: Supplementary file 1 [file Image_1.TIF]

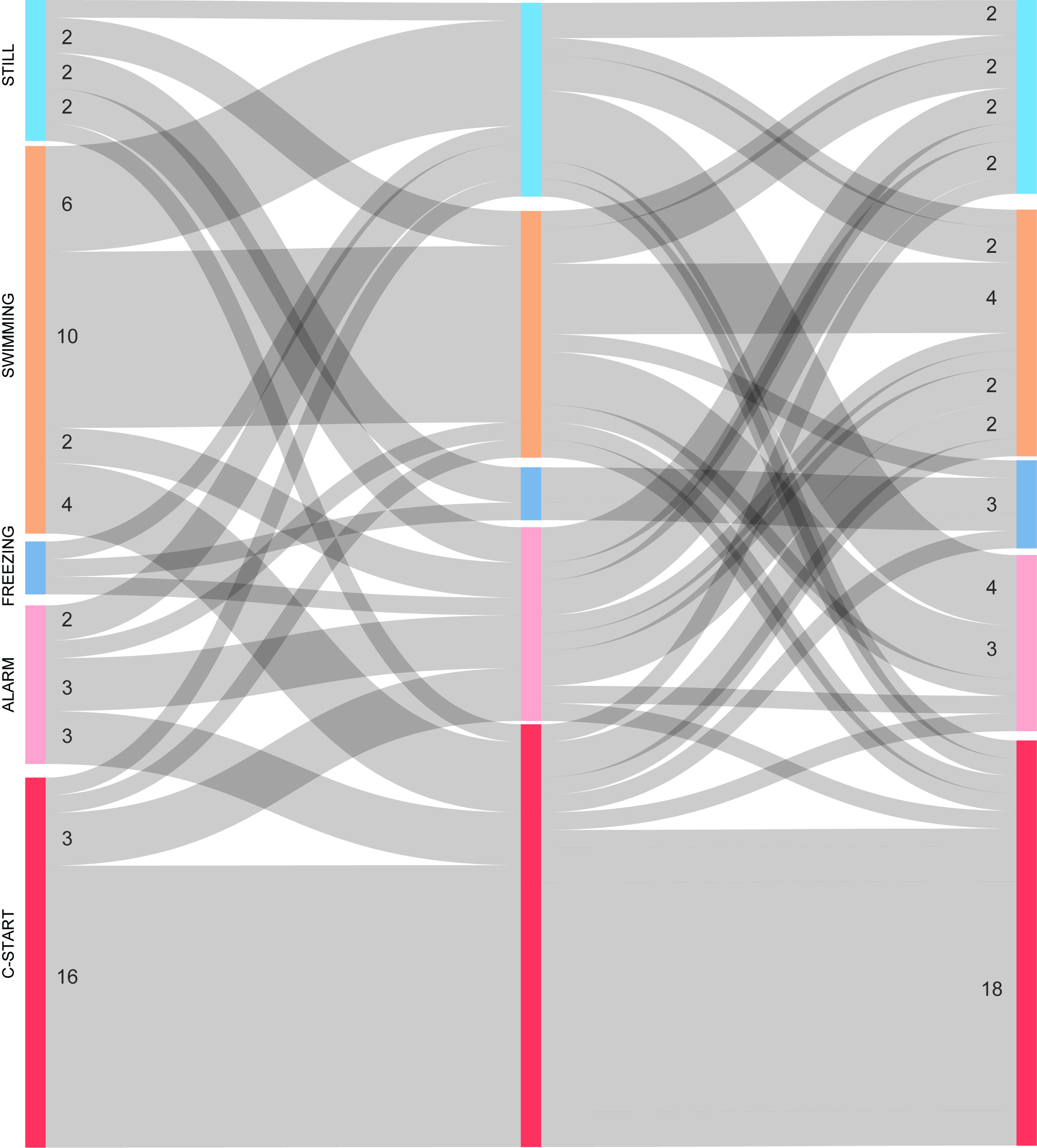

Supplement: Supplementary file 2 [file Image_2.TIF]

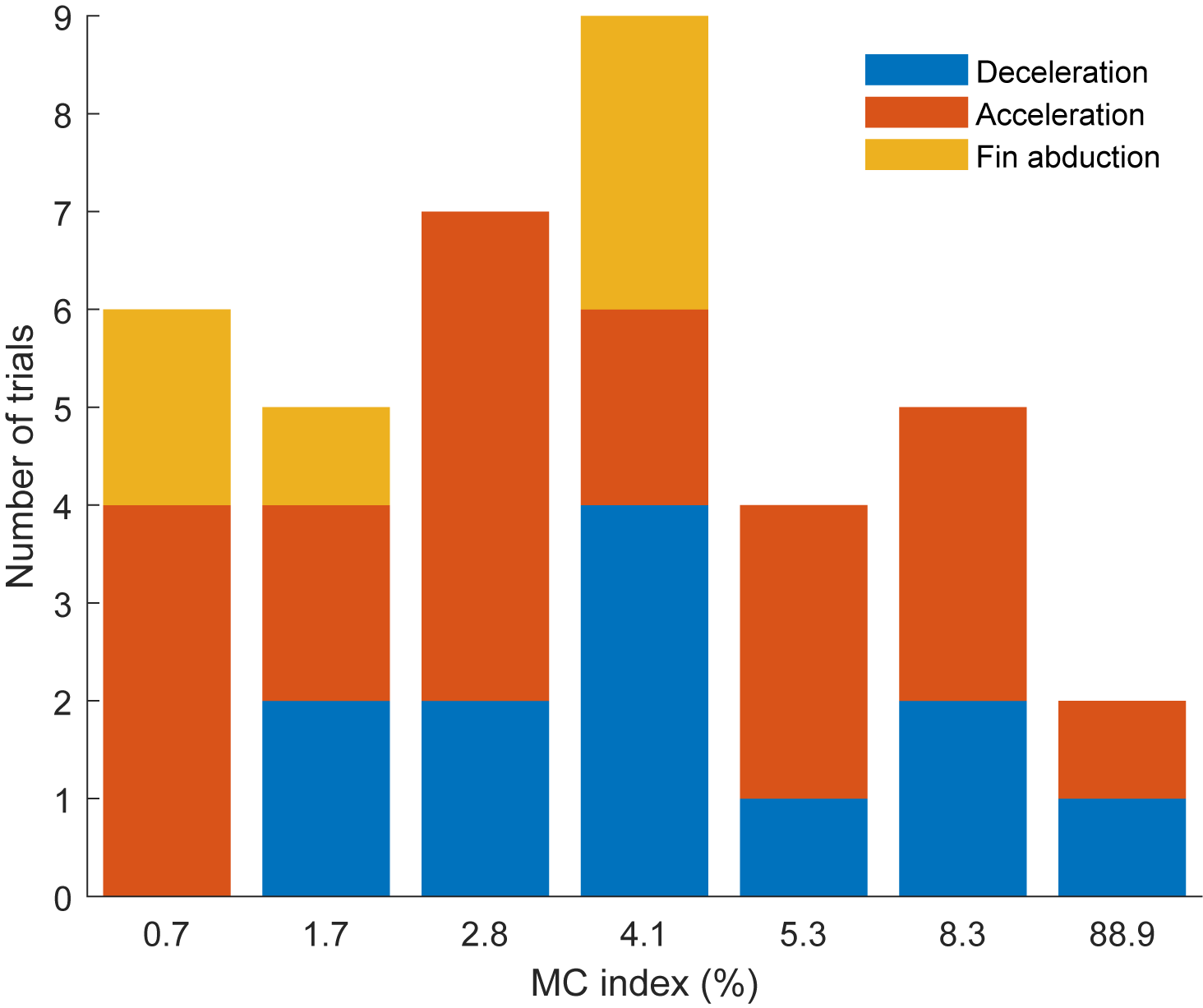

Supplement: Supplementary file 3 [file Image_3.TIF]
