# Supplementary material for: Stimulus Contrast Information Modulates Sensorimotor Decision Making in Goldfish
Source: Front Neural Circuits. 2020 May 28;14:23. doi: 10.3389/fncir.2020.00023 (PMC7270408; doi:10.3389/fncir.2020.00023)
Supplement: Supplementary file 4 [file Table_1.DOCX]

Supplementary Figure Legends

**Supplementary figure 1.** The tile plot represents the type of behavior each animal performed on three consecutive trials of the same stimulus presentation. Grey bars on the left show the contrast of the stimulus animals were stimulated with.

**Supplementary figure 2.** Alluvial diagram of the motor behavior observed after each stimulus presentation. The diagram shows the motor animal’s motor response to the first (left), second (middle) and third (right) looming stimulation. Number of animals that compose each category are indicated within corresponding grey area. Grey areas with no label correspond to 1 animal. The overall proportion of behaviors observed for each trial was similar (Chi-squared test, p = 0.86).

**Supplementary figure 3.** Stacked bars represent the number and type of observed alarm behaviors for each ICS. The categories are based on the descriptions provided in the Methods - Data analysis section, with the Acceleration category also including darting, erratic movements and zig-zagging. The proportion of each class of alarm behavior did not seem to change across ICS (Chi-squared test, p = 0.48).
